# Six- and seven-dimensional experiments by combination of sparse random sampling and projection spectroscopy dedicated for backbone resonance assignment of intrinsically disordered proteins

**DOI:** 10.1007/s10858-015-9987-7

**Published:** 2015-09-24

**Authors:** Szymon Żerko, Wiktor Koźmiński

**Affiliations:** Faculty of Chemistry, Biological and Chemical Research Centre, University of Warsaw, Żwirki i Wigury 101, 02089 Warsaw, Poland

**Keywords:** Intrinsically disordered proteins, Resonance assignment, High-dimensionality NMR, Non-uniform sampling, Projection spectroscopy

## Abstract

Two novel six- and seven-dimensional NMR experiments are proposed. The new experiments employ non-uniform sampling that enables achieving high resolution in four indirectly detected dimensions and synchronous sampling in the additional dimensions using projection spectroscopy principle. The resulted data sets could be processed as five-dimensional data using existing software. The experiments facilitate resonance assignment of intrinsically disordered proteins. The novel experiments were successfully tested using 1 mM sample of α-synuclein on 600 and 800 MHz NMR spectrometers equipped with standard room temperature probes. The experiments allowed backbone assignment from a 1-day acquisition.

## Introduction

In recent years, intrinsically disordered proteins (IDPs) have attracted widespread interest in molecular biology research (Wright and Dyson 1999; Habchi et al. 2014). Their properties like structural propensity, dynamics and interactions could be effectively studied by solution NMR spectroscopy methods. However, the intrinsic disorder results in a fast conformational dynamics which causes an effective averaging of chemical shifts. Thus, very poor peak separation makes resonance assignment difficult, even for a relatively small disordered protein fragments. As a consequence, the signal overlap in the spectra strongly limits the possibility of standard NMR experiments to provide sufficient resolution for IDPs characterization. On the other hand, the fast local dynamics significantly decreases transverse relaxation rates, enabling the application of long multidimensional pulse sequences, and making possible to achieve long evolution times in order to increase the resolution and facilitate assignment of resonances.

The projection NMR spectroscopy methods are founded on the idea of Accordion Spectroscopy (Bodenhausen and Ernst 1981), and employ synchronous incrementing of two or more evolution periods in the pulse sequence. The particularly important approach of this type is simultaneous sampling of more than one chemical shift evolution, which is referred to as radial sampling. Such an option is utilized in projection spectroscopy (Kupče and Freeman 2005; Coggins et al. 2004, 2010), and requires the algebraic decoding of peak frequencies (Kim and Szyperski 2003; Koźmiński and Zhukov 2003; Hiller et al. 2005), or the reconstruction of multidimensional spectrum (Kupče and Freeman 2003).

To date, several high dimensional strategies have been proposed for backbone resonance assignment of IDPs (Reddy and Hosur 2014; Goradia et al. 2015; Yoshimura et al. 2015), including direct ^13^C detection (Bermel et al. 2006, 2009; Nováček et al. 2011; Pantoja-Uceda and Santoro 2013, 2014) and HA detection (Mäntylahti et al. 2010, 2011; Yao et al. 2014) which allow for revealing of proline residues. The number of dimensions, i.e. number of simultaneously correlated frequencies, could be increased by employing automated projection spectroscopy (APSY) (Hiller et al. 2007; Narayanan et al. 2010; Yao et al. 2014), or the non-uniformly sampled (NUS) high-dimensional experiments (Kazimierczuk et al. 2010; Zawadzka-Kazimierczuk et al. 2012).

APSY approach enabled the acquisition of experiments with dimensionality up to 7, by recording a set of 2D projections of high dimensionality spectrum at different angles. The high-dimensional peak list is achieved by algebraic reconstruction from automatically collected peak coordinates in the projections. The possible disadvantage of this technique is severe peak overlap on 2D spectral projections, which may influence the precision of frequency determination.

The advantage of non-uniformly sampled high-dimensionality experiments, followed by SMFT (sparse multidimensional Fourier transform) processing (Kazimierczuk et al. 2009), is the ability for visual inspection of the spectrum of the full dimensionality. Such examination of 2D cross-sections helps to assign peaks suffering from partial overlap.

SMFT processing procedure with the output in the form of a series of 2D cross-sections, requires, however, a so-called base spectrum of dimensionality of N-2, which becomes impractical for N > 5. Therefore, we propose here new 6 and 7D experiments that feature high resolution and high dimensionality resulting from the use of non-uniform sampling in the indirectly detected dimensions combined with projection spectroscopy principle. The experiments utilize NUS in four indirectly sampled dimensions, while additional frequencies are added by synchronous incrementing with *t*_1_. Thus, the previously described 5D SMFT processing with a 3D base spectrum could be used for the proposed experiments, resulting in a 5D spectrum with additional dimensions projected into *F*_1_.

Since the N and CO chemical shifts were found to be best dispersed in IDPs (Piai et al. 2014; Nowakowski et al. 2015), the proposed experiments are derived from 5D (H)NCO(NCA)CONH (Zawadzka-Kazimierczuk et al. 2012), by adding H^N^ (6D) or H^N^ and CA (7D) chemical shifts. The experimental verification was performed on a 1 mM sample of α-synuclein, employing 600 and 800 MHz NMR spectrometers with standard RT triple resonance probes.

## Materials and methods

### Pulse sequence

**Fig. 1:**
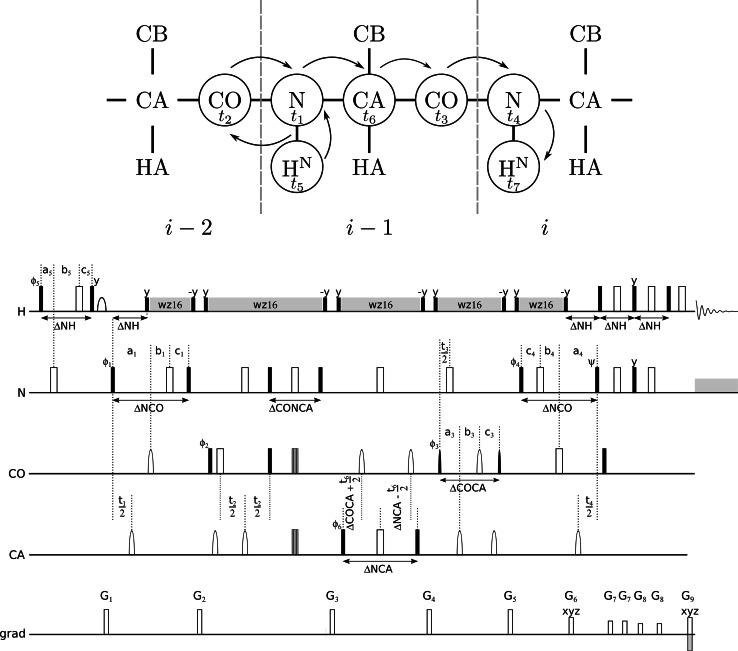
HNCO(N)CACONH experiment. *Top scheme* shows the coherence pathway involved in the experiment. *Bottom* is the pulse sequence scheme. *Rectangles* represent hard pulses. *Filled* and *empty symbols* represent 90° and 180° pulses, respectively. ^1^H and ^15^N composite pulse decouplings are performed with WALTZ-16 (Shaka et al. 1983), at γB_1_/2π of 5.4 and 1.14 kHz at the 800 MHz spectrometer, and 4.0 and 0.97 kHz at 600 MHz, respectively. Simultaneous inversion of CA and CO spins was archived using 6-element composite pulse (Shaka 1985). Selective CA and CO ^13^C 90° (180°) pulses were applied with rf field strength adjusted to |ΔΩCA-CO|/√15 (√3). At the 800 MHz spectrometer 90° and 180°, rectangular and sinc-shaped pulses (bell-shaped at the scheme) of the duration of 40.1 (35.9) μs, and 65.8 (58.8) μs, respectively, were used. Whereas, at the 600 MHz spectrometer of, 53.5 (47.9) μs, and 87.8 (78.4) μs, respectively. Off-resonance pulses were applied using phase modulation of the carrier. The amplitude (Tm^−1^) of G_1_–G_9_ PFG pulses were set to: 0.212, 0.154, 0.137, 0.0926, 0.0820, 0.347, 0.154, 0.0579, 0.352 at the 800 MHz spectrometer and at 600 MHz: 0.222, 0.162, 0.143, 0.0970, 0.0858, 0.364, 0.162, 0.0606, 0.368, respectively. The PFG duration of G_1_–G_5_, G_8_ of 0.5 ms, G_6_ and G_7_ of 2.0 ms and G_9_ of 0.2 ms, were used. Evolutions for H, N, CA were in semi-constant-time mode: *a*_*i*_ = (*t*_*i*_ + Δ)/2; *b*_*i*_ = *t*_*i*_(1 − Δ/*t*_imax_)/2; *c*_*i*_ = Δ(1 − *t*_*i*_/*t*_imax_)/2 or in constant-time mode: *a*_*i*_ = (*t*_*i*_ + Δ)/2; *b*_*i*_ = 0; *c*_*i*_ = (Δ − *t*_*i*_)/2 where Δ stands for ΔN–H, ΔN–CO, ΔN–CA. Evolution for CO in *t*_2_ is in real-time mode. Delays were set as follows: ΔN–H = 5.4 ms, ΔN–CO = 28 ms, ΔCO–N–CA = 28 ms, ΔN–CA = 54 ms, ΔCO–CA = 9.1 ms. The four step phase cycle was used: ϕ_1_ = x, -x; ϕ_2_ = 2x, 2(-x) and ϕ_rec_ = x, 2(-x), x = ϕ_1_ + ϕ_2_. In *t*_1_, *t*_2_, *t*_3_, *t*_4_ dimensions quadrature was accomplished using States-TPPI method, by incrementing ϕ_1_, ϕ_2_, ϕ_3_, ϕ_4_ phases, respectively. In *t*_5_, *t*_6_ dimensions quadrature was accomplished using States method by incrementing ϕ_5_, ϕ_6_ phases and adding additional increments to phase ϕ_1_ = ϕ_1_ + ϕ_5_ + ϕ_6_, thus, the ϕ_1_ phase was incremented by additional 90° for each sine modulation in *t*_1_, *t*_5_ and *t*_6_. The phase ψ = x was inverted simultaneously with the last gradient (G_9_) pulse to achieve echo-antiecho coherence transfer selection in the indirect dimension. The coherence selection gradients (G_6_ and G_9_) were applied at magic angle (600 MHz) or along z-axis (800 MHz). 90° and 180° water 1.2 ms *sinc-shaped* flipback pulses were used for ϕ_5_ phase equal to x and y, respectively. For 5D HNCO(N)CACONH experiment *t*_5,_ *t*_6_ and ϕ_5_, ϕ_6_ were set to 0 s and x, respectively. Additional dimensions are achieved by setting evolution times (*t*_5_ and/or *t*_6_) proportional to *t*_1_. The ratio of maximum evolution times determined the respective projection angle. Note, that two different 6D and one 7D experiments could be acquired according to presented scheme

The proposed pulse sequence is based on 5D (H)NCO(NCA)CONH presented in (Zawadzka-Kazimierczuk et al. 2012) and is depicted in the Fig. 1.

The original pulse sequence was designed to obtain both sequential and auto-correlation peaks by setting ΔN–CA delay to 28.6 ms. It surely allows easier visual inspection of the spectra. On the other hand, the doubled number of peaks increases sampling artefacts level. Moreover, lack of auto-correlation peaks present in the spectrum reduces spectral overlap, which may affect accuracy of measured peaks positions. This is especially important while studying IDPs. The coherence transfer amplitudes are described by Eqs. (1) and (2):

1 $$I_{auto} \propto \cos \left( {{}_{{}}^{1} J_{NCA} \pi t} \right)\cos \left( {{}_{{}}^{2} J_{NCA} \pi t} \right)\cos \left( {{}_{{}}^{1} J_{CACB} \pi t} \right)$$

2 $$I_{seq} \propto - \sin \left( {{}_{{}}^{1} J_{NCA} \pi t} \right)\sin \left( {{}_{{}}^{2} J_{NCA} \pi t} \right)\cos \left( {{}_{{}}^{1} J_{CACB} \pi t} \right)$$

**Fig. 2:**
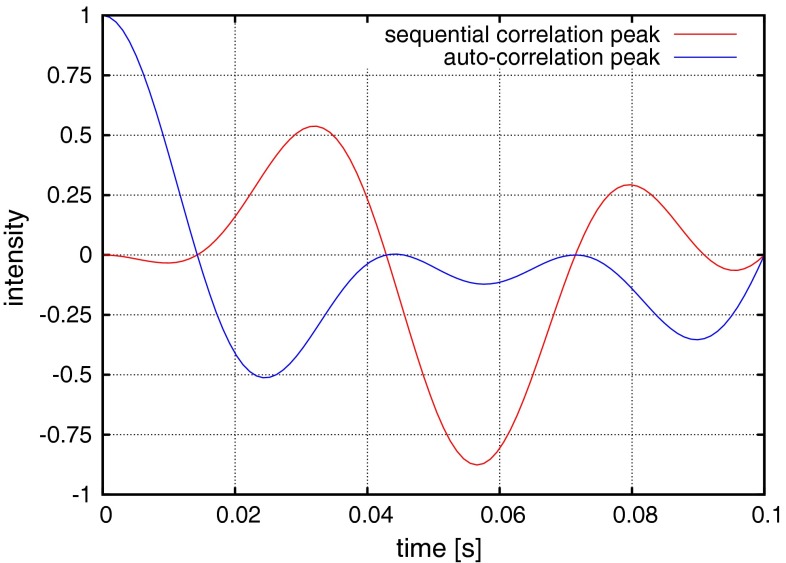
Transfer efficiencies for auto-correlation and sequential peaks as a function of ΔN–CA delay length

Assuming ${}_{{}}^{1} J_{NCA} ,{}_{{}}^{2} J_{NCA}$, ${}_{{}}^{1} J_{CACB}$ values of 11, 7 and 35 Hz (Sattler et al. 1999), respectively, the optimal choice of ΔN–CA delay length for both high intensity of sequential signal (N_i−1_(ω_1_), CO_i−2_(ω_2_), CO_i−1_(ω_3_), N_i_(ω_4_), H_i_^N^(ω_5_)) and the suppression of auto-correlation signal (N_i_(ω_1_), CO_i−1_(ω_2_), CO_i−1_(ω_3_), N_i_(ω_4_), H_i_^N^(ω_5_)) can be obtained with the value of 54 ms (see Fig. 2), similar approach was also presented by Nováček et al. 2011. In the present work the auto-correlation peaks suppressed version was used.

Quadrature for *t*_1_ dimensions is achieved using a standard States-TPPI procedure. Quadratures in coevolved dimensions require recording and storing of two data sets, cosine and sine modulated, respectively. It is obtained by 90 degree shift of phases ϕ_5_, and ϕ_5_, ϕ_6_ (for 6D and 7D versions respectively) (Koźmiński and Zhukov 2003; Kim and Szyperski 2003). To allow standard SMFT data processing the π/2 phase shifts for sine modulated components are compensated by simultaneous π/2 shifting of ϕ_1_ phase, for *t*_1_ dimension. Therefore, such procedure of processing of spectra after SMFT approach is limited to a simple co-addition to obtain spectra differing in frequency signs in coevolved dimensions.

### Data processing and inspection

**Fig. 3:**
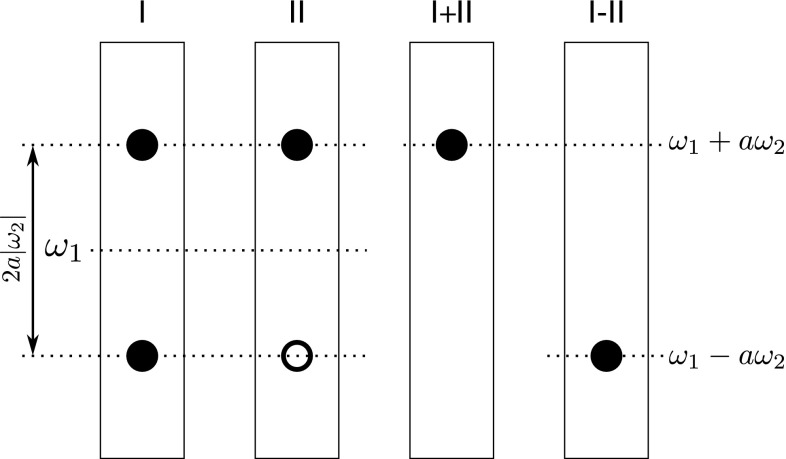
Pictorial representation of resulting peak pattern when two dimensions are coevolved. Two modulations lead to spectra I and II containing 2 peaks each. Sum and difference of obtained spectra gives two different spectra containing only one peak each, for which frequencies are a linear combinations of frequencies from coevolved dimensions. *a* coefficient is equal to *t*_2_/*t*_1_. *Empty* and *filled circles* represent positive and negative signal intensities, respectively

**Fig. 4:**
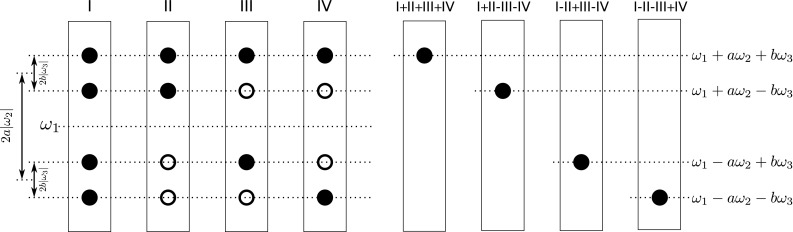
Pictorial representation of resulting peak pattern when three dimensions are coevolved. Four modulations lead to spectra I, II, III and IV containing 4 peaks each. Co-addition of obtained spectra gives four different spectra containing only one peak each, for which frequencies are a linear combinations of frequencies from coevolved dimensions. *a*, and *b* coefficients are equal to *t*_2_/*t*_1_, and *t*_3_/*t*_1_, respectively. *Empty* and *filled circles* represent positive and negative signal intensities, respectively

The presented experiments are, in fact, projections of six- and seven-dimensional experiments to the five- dimensional frequency space. Therefore, both of the presented experiments were processed as a usual 5D spectrum using SMFT approach. Last three dimensions (CO, N, H^N^) were fixed with chemical shifts obtained from 3D HNCO experiment. This lead to a set of 2D cross-sections showing correlations of coevolved dimensions (*t*_1_, *t*_5_, *t*_6_) with CO (*t*_2_). A simple co-addition of differently modulated data sets result with two data sets (in a case of 6D), which differ with signs of involved frequencies (7D version results in four different combinations), this procedure is schematically illustrated in the Figs. 3 and 4.

All 5D spectra were processed using ToASTD (Kazimierczuk et al. 2006) and reduced (Kazimierczuk et al. 2009) programs which take peak list from 3D HNCO as the input for SMFT routine. For 3D HNCO sampling artefacts were removed from the spectra using Signal Separation Algorithm (Stanek and Koźmiński 2010), no such procedure was performed for 5D spectra. All used programs are available at http://nmr.cent3.uw.edu.pl/software. After processing, all spectra were inspected using Sparky (Goddard and Kneller 2000).

Four distinct peaks frequencies in the projected dimension: $\Omega_{1}$, …, $\Omega_{4}$ on the transformations for 6D experiment are given by the Eq. (3):

3 $$\Omega_{1,2,3,4} = \pm \Omega_{N} \pm \Omega_{H} \frac{{t_{5} }}{{t_{1} }}$$

In the case of 7D data the analogous expressions for peak frequencies: $\Omega_{1}$, …, $\Omega_{8}$ are given by the Eq. (4)

4 $$\Omega_{1,2,3,4,5,6,7,8} = \pm \Omega_{N} \pm \Omega_{H} \frac{{t_{5} }}{{t_{1} }} \pm \Omega_{CA} \frac{{t_{6} }}{{t_{1} }}$$

$\Omega_{N}$,$\Omega_{H}$, $\Omega_{CA}$ are the values of resonance frequencies of N, H and CA nuclei of the $n - 1$ residue with respect to the corresponding carrier offsets. $t_{1}$, $t_{5}$, $t_{6}$ are maximum evolution times in N, H and CA dimensions, respectively. As a result, for 6D spectrum, a system of four equations (two of them are linearly independent) with two unknown is obtained. In the case of 7D spectrum system of 8 equations (four linearly independent) with three unknown is created. Solving these systems of equations results in $\Omega_{N}$, $\Omega_{H}$, $\Omega_{CA}$ frequencies. The first system of equations (Eq. 3) is unambiguously defined, however, the second one (Eq. 4) is overdetermined. Three equations are sufficient to solve it but in order to increase the precision of a result, all four equations with equal weights were always used.

### NMR spectroscopy

NMR sample contained 1 mM of ^13^C, ^15^N-labeled α-synuclein in 20 mM sodium phosphate buffer, pH 6.5, 200 mM NaCl. NMR spectra were recorded at 288 K on Agilent 800 MHz and 600 MHz spectrometers both equipped with room temperature probes. 7D HNCO(N)CACONH was acquired on the 800 MHz spectrometer in 31 h with evolution times set to 20, 30, 25, 52, 25, 30 ms for H_i_^N^, N_i_, CO_i−1_, CA_i−1_, CO_i_, N_i+1_ dimensions, respectively. 6D HNCO(NCA)CONH was acquired on both, 800 MHz and 600 MHz, spectrometers in 13 and 23 h, respectively. Evolution times for the measurement on the 800 MHz spectrometer were set to: 20, 30, 25, 25, 30 ms, for H_i_^N^, N_i_, CO_i−1_, CO_i_, N_i+1_ dimensions, respectively, and to sustain high enough resolution on the lower field evolution times were set to: 20, 40, 40, 40, 40 ms, respectively on the 600 MHz spectrometer.

## Results

In order to verify results obtained from 6D and 7D projection experiments full backbone assignment of the α-synuclein was performed using 5D HN(CA)CONH (Kazimierczuk et al. 2010), 5D (H)NCO(NCA)CONH (Zawadzka-Kazimierczuk et al. 2012) and 5D HabCabCONH (Kazimierczuk et al. 2010) experiments. To avoid any discrepancies, exactly the same sample as for 6D and 7D measurements was used.

Analysis of both recorded 6D spectra led to identification of all but two expected sequential peaks. Two missing residues were the first two residues in the protein sequence which suggest that those signals were broadened beyond detection limit due to amide proton exchange. Similar result was obtained from 7D spectrum, the same set of sequential signals was identified.

**Fig. 5:**
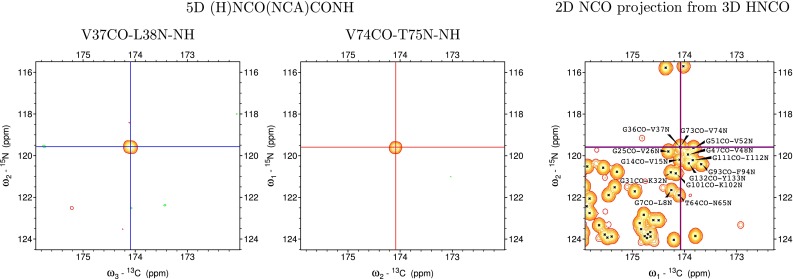
Two 2D cross-sections from 5D (H)NCO(NCA)CONH experiment (*on the left*) corresponding to HNCO peak positions of G36CO-V37N and G73CO-V74N signals showing CO_i−1_N_i_ sequential peaks. 2D NCO projection from 3D HNCO (*on the right*) showing highly crowded glycine region. Unambiguous assignment is prevented due to signal overlap

**Fig. 6:**
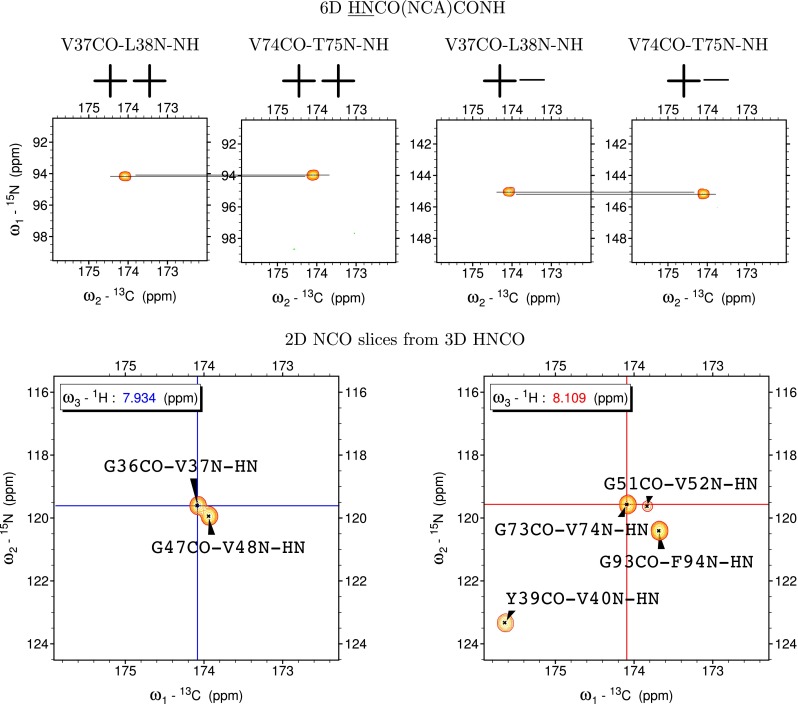
Four 2D cross-sections from 6D HNCO(NCA)CONH experiment (*at the top*) (++ and +− corresponds to the signs in the co-addition of obtained raw data sets) corresponding to the HNCO peak positions of G36CO-V37N and G73CO-V74N signals showing CO_i−1_(N_i_ H_i_^N^) sequential peaks. Small difference in frequencies in the measured peak positions encodes additional information of H^N^ chemical shifts. Two slices from 3D HNCO spectra obtained at H^N^ the positions calculated from 6D experiment data (*at the bottom*). Unambiguous assignment is possible owing to the difference in H^N^ chemical shifts of V37 and V74. Note that *y*-axis at the cross-sections from the 6D HNCO(NCA)CONH spectrum is labelled with ^15^N chemical shift scale, however, the peak frequencies in this dimension are given by the Eq. (3)

**Fig. 7:**
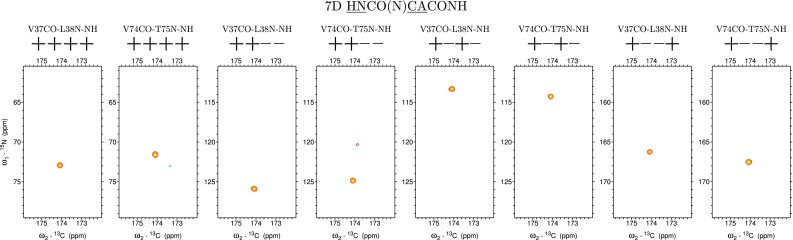
Eight 2D cross-sections from 7D HNCO(N)CACONH experiment (++++ , ++−, +−+− and +− + correspond to the signs in the co-addition of obtained raw data sets) corresponding to the HNCO peak positions of G36CO-V37N and G73CO-V74N signals showing CA _i−1_CO_i−1_(N_i_ H_i_^N^) sequential peaks. While coevolution of H^N^ leads to a more robust connectivity adding of CA frequency further differentiate signal positions due to the difference in CA chemical shifts of V37 and V74. Note that *y*-axis at the cross-sections from the 7D HNCO(N)CACONH spectrum is labelled with ^15^N chemical shift scale, however, the peak frequencies in this dimension are given by the Eq. (4)

Performing sequential assignment of α-synuclein due to its disordered nature can be regarded as a challenging task. Despite α-synuclein’s moderate size (140 a.a.), full potential of the “HNCO-HNCO” strategy can be seen, for example, in the case of G36CO-V37N and G73CO-V74N signals which are hardly distinguishable using CO_i−1_N_i_ connectivity (see Fig. 5). Application of 6D experiment quickly resolves such ambiguity without using any additional experiment as the H^N^ chemical shifts of aforementioned residues vary by almost 0.2 ppm (see Fig. 6). Moreover, peak positions in the resulting spectra are further differentiated when seventh dimension (CA_i−1_) is introduced (see Fig. 7). In addition to a better signal dispersion CA chemical shift provide partial information about residue type.

## Discussion

The most important advantage of presented approach is the establishment of highly robust connectivities due to the expansion of sequential peaks frequencies by addition of H_i−1_^N^ frequency. Similar approach was already proposed in the literature which involved the combination of two different 5D experiments (Kazimierczuk et al. 2013; Piai et al. 2014) having shared sequential nitrogen dimension. Here it was presented that such “HNCO-HNCO” strategy can be successfully implemented within one experiment. Additional expansion to 7D experiment giving CA chemical shift can further limit number of overlapping signals as well as provide partial information needed for identifying amino acid type.

True benefit of proposed approach is the possibility of a visual inspection of resulting spectra which allows to use the expertise and the experience of a spectroscopist in resolving most difficult, severely overlapped cases. As a result, all of non-proline residues of α-synuclein were successfully identified and assigned from a single experiment (except aforementioned first two residues).

Surprisingly, even example of a moderate size α-synuclein already justifies expanding “CON-CON” strategy. Presented approach can be especially beneficial in performing the resonance assignment of proteins of a larger size than α-synuclein, since the spectra in such a case will be more crowded. What is more, sensitivity of presented experiments is high enough to record good quality spectra in a relatively short time compared to other six- and seven-dimensional approaches (Hiller et al. 2007; Yao et al. 2014), even without the use of cryogenically cooled probe.

We have demonstrated the principles of the new experiment and data inspection protocol. Although, it could be modified in several ways. The proposed experiment is fully compatible with BEST approach (Lescop et al. 2007; Solyom et al. 2013) and co-solute paramagnetic relaxation enhancement (Oktaviani et al. 2015), which could be used for acceleration of the signal repetition rate, and thus increase the number of data points acquired in a given time. Amide hydrogen exchange process, particularly effective in the IDP molecules, may cause a severe signal loss at conditions close to physiological (temperature and pH). Therefore, the pulse sequence could be modified using the aliphatic proton excitation combined with the ^13^C or HA detection (Mäntylahti et al. 2010). Moreover, aliphatic protons or ^13^C detection would allow observation of additional cross-peaks from proline residues (Bermel et al. 2006; Hellman et al. 2014). Additionally, the amide protons exchange causes further decay of ^15^N–^1^H antiphase coherences. Hence, the refocusing of ^15^N–^1^H couplings and ^1^H composite pulse decoupling applied during the main part of pulse sequence is used to maximize the sensitivity.

## Conclusions

We presented six- and seven-dimensional experiments for backbone assignment of intrinsically disordered proteins. Described techniques combine projection spectroscopy and SMFT data processing that allow access to full dimensional spectra information. 6D experiment correlates H_i_^N^, N_i_, CO_i−1_, H_i−1_^N^, N_i−1_, CO_i−2_. 7D adds additional correlation with CA_i−1_ dimension. We proved that these experiments can be successfully run on a standard room-temperature probe, even on a medium field NMR spectrometer (600 MHz).
